# Comparison of the Accuracy of CBCT Images and Apex Locator in Detection of External Root Resorption with Perforation

**DOI:** 10.30476/DENTJODS.2021.91056.1548

**Published:** 2022-12

**Authors:** Afsoon Adibi, Fereshteh Sobhnamayan, Nazafarin Ostovar Zijerdi, Mohammad Tajik, Maryam Paknahad

**Affiliations:** 1 Dentist, School of Dentistry, Shiraz University of Medical Sciences, Shiraz, Iran; 2 Dept. of Endodontics, School of Dentistry, Shiraz University of Medical Sciences, Shiraz, Iran; 3 Student of Research Committee, Dental School, Shiraz University of Medical Sciences, Shiraz, Iran; 4 Postgraduate Student, Dept. of Oral and Maxillofacial Radiology, School of Dentistry, Shiraz University of Medical Sciences, Shiraz, Iran; 5 Oral and Dental Disease Research Center Dept. of Oral and maxillofacial Radiology, School of Dentistry, Shiraz University of Medical Sciences, Shiraz, Iran

**Keywords:** Cone-Beam Computed Tomography, Dental pulp cavity, Tooth Preparation, Tooth Resorption

## Abstract

**Statement of the Problem:**

Perforation within external root resorption (ERR) lesions dra-matically affects the prognosis of the involved roots. Failure to diagnose perforation under-mines treatment; therefore, early detection of these lesions is of great importance. The cone-beam computed tomography (CBCT) images and electronic apex locators (EAL) are reliably used to detect root perforations.

**Purpose:**

In this in vitro study, we compared the EAL findings with the results obtained by the CBCT images for the detection of perforations within ERR lesions.

**Materials and Method:**

This cross sectional study included 160 extracted anterior human teeth. The teeth were categorized into four groups including teeth with intact root, teeth with ERR, teeth with ERR and 0.5 mm perforation, and teeth with ERR with 1 mm perforation. The presence of perforations was compared by CBCT images and root EAL.

**Results:**

The sensitivity of CBCT scans in detecting 0.5 mm and 1 mm root perforations was 100% and 97.5%, respectively, while the sensitivity of the EAL was 10% and 27.5% in de-tecting 0.5 mm and 1 mm root perforations, respectively( with the ±0.5mm range of error). For detecting intact and teeth with ERR, the specificity of CBCT scans was 100% and 95%, respectively, and for EAL, it was 100% for both. Poor agreement was found between the two techniques (kappa=-0.025).

**Conclusion:**

CBCT scans were more sensitive and specific than EAL scans for detecting perforations in non-obturated root canals in this study.

## Introduction

Periodontium is exposed to the oral cavity by root perforation (RP), a mechanical or iatrogenic communication between the root canal space and the periodontal apparatus [ 1 ]. The supporting tissues of the tooth become contaminated by bacteria as a result [ 1 - 2 ]. RP may happen due to internal or external root resorption (ERR) [ 3 ]. The condition also affects 2% to 12% of endodontically treated teeth and accounts for 10% of periradicular tissue failures [ 3 ]. The prognosis of root perforations is significantly affected by the location, size, and time [ 4 ]. Repairing large perforations may be more difficult than repairing smaller ones. Small perforations have a good chance of healing predictably. In addition, early detection and proper management are associated with prolonged survival rates [ 5 ]. 

Several devices and techniques have been suggested for detecting perforations, such as periapical radiography, electronic apex locators (EAL), operative microscopes, endoscopes, computed tomography, and cone-beam computed tomography (CBCT). The use of CBCT in the oral and maxillofacial region is a new advancement [ 6 - 7 ]. Previous studies have shown that CBCT is the most accurate imaging modality for detecting perforations when compared to conventional intraoral radiography, photostimulable phosphor plates, and multidetector computed tomography [ 8 - 10 ]. There is also evident that CBCT provides a diagnostic accuracy of 81.3 % in detecting micro perforations within internal root resorption lesions [ 11 ]. 

One of the other common devices, which are utilized in the root-perforation diagnosis, is EALs [ 12 ]. Previous studies [ 13 - 14 ] compared the diagnostic accuracy of various EALs. These devices provide excellent accuracy in locating root perforations. 

It has been reported that enlarging the size of the perforation will result in the increase of sensitivity rates of EALs in the detection of simulated root perforations [ 8 ]. As mentioned before, perforations with a smaller size and earlier diagnosis at the time of detection have a better prognosis. For the best understanding of this issue, a few studies [ 2 , 15 - 16 ] investigated the agreement between two accurate and commonly used techniques including CBCT and EALs. Therefore, this study was designed to compare CBCT and EAL accuracy in detecting root perforations.

## Materials and Method

**Figure 1:**
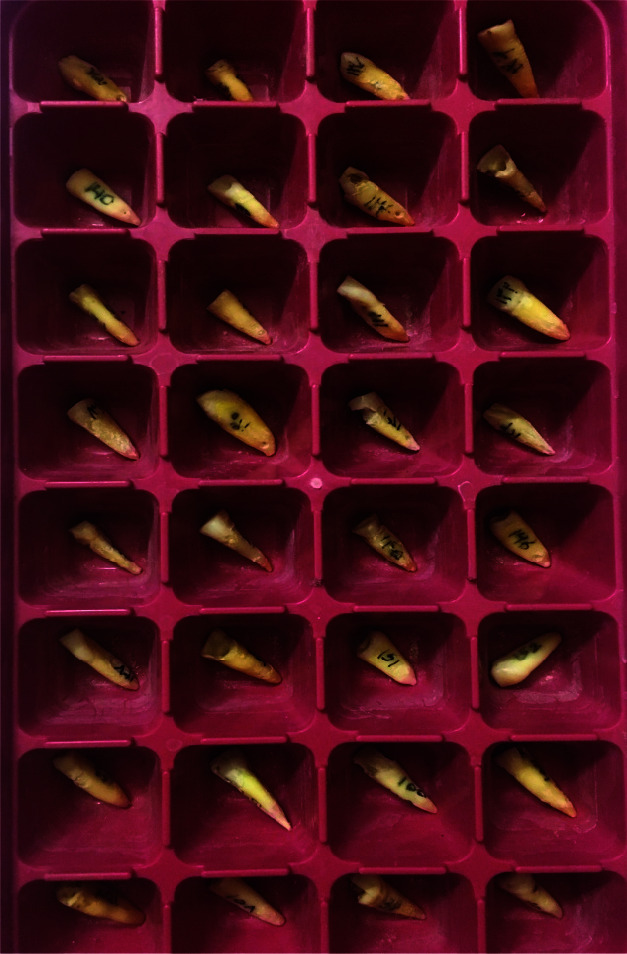
The teeth with single root canals, ≥1.5-mm dentin wall thickness in the middle third of the root and no caries were selected for this study

The present study was approved by the Shiraz university of the ethics committee (IR.SUMS.REC.1396. S110). 160 extracted, single-root, anterior human teeth (extracted for periodontal or orthodontic reasons) were selected (Figure 1). The selection criteria included the teeth with single root canals, ≥1.5-mm dentin wall thickness in the middle third of the root and no caries, restoration, root filling, pathosis, or anomaly, with mature apices and straight roots. The study excluded teeth with root fractures, obstructed apexes, previous root perforations, and calcified canals. This selection was achieved through obtaining a preliminary periapical radiograph and direct observation of the teeth. The samples were taken from the individuals who had given the consent for their extracted teeth to be used for the research purposes. 

### Tooth preparation

The samples were initially stored in distilled water containing 10% formalin and kept refrigerated. All calculus and residual organic debris on the outer surfaces of the roots were removed by ultrasonic device (EMS Piezon® Master 600/Nyon, Switzerland).

To produce stable reference points, each tooth crown was flattened using a tapered diamond bur (D&Z, Switzerland) and a high-speed hand-piece. Standard access cavity was prepared, and the canal patency was confirmed. By using a stereomicroscope (Zeiss Stemi, Carl Zeiss/ GmbH, Germany) under a magnification of 15 ×under multipower illumination, the actual length of the canal was determined by using a K-file size 10 (Mani Co., Japan). A working length of 0.5 mm less than this was considered acceptable. The canal's contents were removed with conventional K-type files that matched the canal's diameter.

Using #2 and #3 Gates Glidden drills (Dentsply Maillefer, Ballaigues, Switzerland), the canal's coronal preflaring was passively performed. The apical patency was maintained via insertion of a K-file size 8 through the foramen during canal instrumentation. 

Teeth were categorized into four groups including teeth with intact root, teeth with ERR, teeth with ERR and 0.5mm perforation, and teeth with ERR with 1 mm perforation. To simulate the actual clinical condition, perforations were made within the ERR lesions. In accordance with a protocol reported in the literature, 2.1 mm cavities were drilled in plaster bases for the teeth [ 10 , 13 , 17 - 19 ]. The cavities were located at the distal surface, 4 mm above the apical foramen. An adapted device for measuring cavities' diameter and depth ensured accuracy of drilling with a #7 high-speed round bur (2.1mm). The simulated ERRs were probed with a dental probe to determine if they communicated with the root canal. Perforations were made with diamond burs fixed in a high-speed handpiece under water coolant as a K-file size 20 was held at working length into the canals until the file was met. The round burs # 1/4 and #2 were employed to produce perforations with diameters of 0.5 and 1 mm.

### Measurements

**Figure 2:**
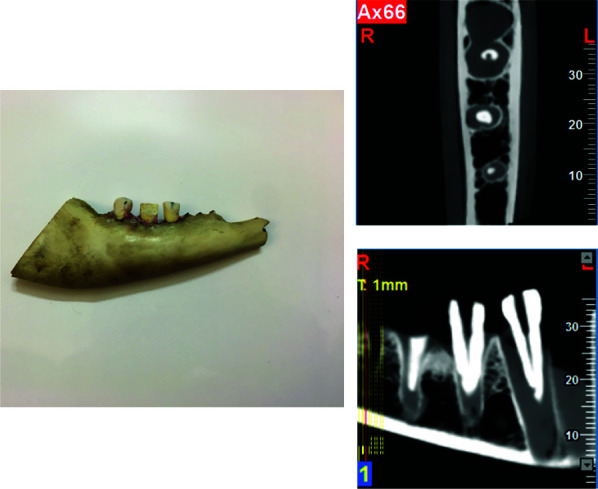
Half-dry sheep mandibles with four teeth, the axial and coronal view of the tooth embedded in wax are shown

For CBCT imaging, half-dry sheep mandibles with four teeth were used (four teeth per mandible). Melted wax was inserted into the sockets prior to tooth insertion. The CBCT examination included the mounting of wax sheets on the labial and lingual surfaces to simulate soft tissue. With a NewTom VGi scanner (NewTom QRsrl, Verona, Italy), CBCT scans were performed at high resolution (voxel size = 0.1mm, field of view = 6×6). CBCT images of teeth showing ERR and perforations were analyzed by an oral and maxillofacial radiologist. Two separate sessions were conducted using a low-lit room with a monitor (18/5-inch Flatron; LG, Seoul, Korea) in order to assess CBCT images in random order. Inter Class Concordance assessment was also performed by an endodontist using CBCT images. Axial and coronal cross-sections of the CBCT data were evaluated (Figure 2). Resorption lesions were projected more clearly using brighter and more contrasted images. During the examination of the teeth, examiners were asked to record whether there was a perforation and to gauge the distance between the perforation site and the most coronal point of the tooth.

An alginate model was created after CBCT images were obtained. An alginate impression material was used on this model as well as plastic (IRALGIN, Golchai Co., Tehran, Iran). We prepared the alginate according to the manufacturer's instructions and packaged it separately. In accordance with the manufacturer's instructions, the root canal was irrigated with sodium hypochlorite at 2.5% and its position was determined. Two board-certified endodontists, who were blinded to both groups and procedures, marked and noted the perforations. The excess sodium hypochlorite from the pulp chamber was removed by cotton pellets. Alginate was used to attach the electrode lip. We gradually introduced a Dentsply Maillefer K-type file of size #15 into the canal. On the screen, the EAL showed '0.0' at the beginning of the root canal perforation, when the micrometer connected to the file was moved apically. A repeatable electronic measurement at 0.0 was performed once after recording the measurement at 0.0, and then the instrument was pulled back to the cervical end (R = 0.0).

### Statistical analysis 

Analyzing the data was done using SPSS software (version 17.0, Chicago, IL, USA). Kappa coefficients were used to assess intra-observer and inter-observer agreement. Sensitivity, specificity, and predictive values were determined for each technique. The level of significance was set at 0.05.

## Results

Using kappa coefficient, high intra- and inter-observer agreements was observed. These results were presented in Table 1. Therefore, the results of the first readings were included for further evaluations. The sensitivity, specificity, positive and negative predictive values for CBCT scans and Root ZX EAL in detecting root perforations are reported in Table 2. 

**Table 1:** The results of Intra and Inter examiner concordance

	Variables	Intra Examiner		Inter Examiner	
		ICC	P	ICC	P
CBCT	Intact	0.873	0.05	0.943	0.05
	ERR	0.863	0.05	0.941	0.05
	RP(0.5mm)	0.843	0.05	0.935	0.05
	RP(1mm)	0.871	0.05	0.940	0.05

CBCT: Cone beam computed tomography, ERR: external root re-sorption, RP: root perforation, P: p value, ICC: intraclass correla-tion coefficient

**Table 2:** The Specificity (Sp), Sensitivity (Sn), Positive Predictive Value (PPV) and Negative Predictive Value (NPV) of diagnosing perforations with ROOT ZX and CBCT (Cone beam computed tomography) Scans

		Sn	Sp	PPV	NPV
CBCT	Intact	-	100%	-	-
	ERR	-	95%	-	-
	RP(0.5mm)	100%	-	95.2%	97.6%
	RP(1mm)	97.5%	-	95.1%	97.4%
EAL (±0.5mm)	Intact	-	100%	-	-
	ERR	-	100%	-	-
	RP(0.5mm)	10%	-	100%	38.1%
	RP(1mm)	27.5%	-	100%	38.1%
EAL (±1mm)	Intact	-	100%	-	-
	ERR	-	100%	-	-
	RP(0.5mm)	17.5%	-	100%	40.8%
	RP(1mm)	37.5%	-	100%	40.8%

CBCT: Cone beam computed tomography, ERR: external root resorp-tion, RP: root perforation, P: p value, ICC: intraclass correlation coefficient

**Figure 3:**
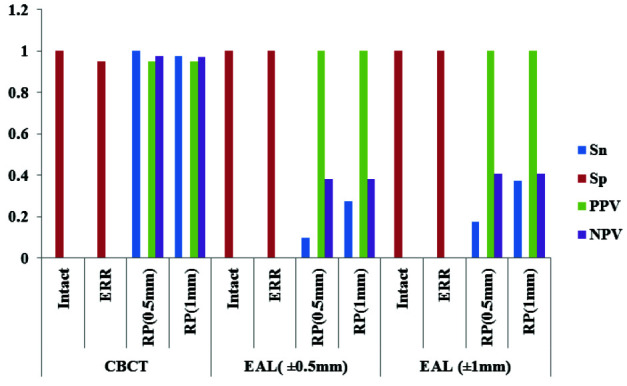
Comparison of the Specificity (Sp), Sensitivity (Sn), Positive Predictive Value (PPV), and Negative Predictive Value (NPV) in diagnosing perforations with Electronic apex locators (EAL) and Cone-Beam Computed Tomograph (CBCT) Scans

In total, 100% (40/40) of 0.5mm RPs and 97.5% (39/40) of the 1mm RPs were detected by CBCT. Considering the ±0.5mm range of error, 10% (4/40) of 0.5mm RPs were detected by the EAL and 27.5% (11/ 40) of the 1mm RPs. These values for the ±1mm range of error were 17.5% (7/40) and 37.5% (15/40) for 0.5mm and 1mm perforations, respectively. For detecting intact and teeth with ERR, the specificity of CBCT scans was 100% and 95%, respectively, and for EAL, it was 100% for both. CBCT scans were overall more significantly accurate than EAL in detecting perforations (Figure 3). Poor agreement was found between the two techniques for detecting both 0.5 and 1mm perforations (both Kappa=-0.025).

## Discussion

Pulpal or periodontal inflammation, orthodontic movement, internal bleach, erupting teeth, tumors and procedural errors are some factors that result in ERR lesions [ 11 , 16 ]. Perforation within ERR lesions dramatically affects the prognosis of involved roots [ 12 ]. Larger perforations are more difficult to manage [ 3 , 14 , 20 ] and failure to diagnose perforation undermines the treatment [ 4 ]. Root perforations can be detected by CBCT and EAL images in many previous studies. Many previous studies [ 4 - 5 , 9 , 13 , 18 , 20 - 23 ] supported the efficacy of CBCT images and EALs in the detection of root perforations. Multi-angled periapical radiographs plays an important role in detecting perforations. However, this technique cannot provide accurate information on the true size, location, and architecture of a lesion because of the overlapping anatomic structures, and the rays’ beam angle [ 3 ]. This limitation can be overcome through advanced imaging modalities such as CBCT [ 2 ]. In this in vitro study, we compared the non-invasive EAL findings with the results obtained by the CBCT images in the detection of perforations within ERR lesions.

In this study, CBCT with higher sensitivity and specificity of 95% was found to be a reliable technique for detecting root perforations in ERR lesions. This superiority was better depicted in small-sized perforations (0.5mm) that showed in Table 2.The results also showed that CBCT had a significantly higher accuracy in detecting perforation compared with EAL (Root ZX). Similarly, using Cranex 3D CBCT in detecting RP in molars showed the sensitivity and specificity rates of 92% and 100%, respectively [ 3 ]. Another study [ 2 ] reported the sensitivity and specificity of 97.9 %and 85.4% in detecting root perforations and strip perforations by Cranex 3D. 

An apex locator can be used to diagnose root perforations confidentially, as reported by Marroquín *et al.* [ 24 ]. Based on D'Assuncao *et al.*’s study [ 8 ], with the error range of 0.5mm, the accuracy rate was reported to be 91.4%, 97.1%, and 100% for Root ZX II, Mini apex, Root SW respectively, in the finding the lateral root perforations with 1mm diameter. Despite the finding that perforation size does not affect CBCT accuracy, Shokri *et al.* [ 2 ] and Venskutonis *et al.* [ 25 ] have found the opposite results. Different sample sizes and imaging systems might explain the discrepancy between reports. In clinical situations, further aspects such as observer’s performance, viewing conditions, patient movement, and software specifications of CBCT can influence the diagnosis of root perforations, hence, additional clinical investigation should be performed [ 2 ]. 

Simulated ERR lesions can be found more efficiently in images with smaller voxel sizes, however, this fact in inconsistent with the results obtained by Da Silveira *et al.* [ 26 ]. Nevertheless, in high-resolution CBCT images of endodontically treated teeth, diagnosis of complications is difficult concerning the beam hardening artifacts of solid materials [ 11 , 21 - 22 , 27 ]. Studies show that using voxel sizes <0.2mm increases the image noise and afflicts the observation of low contrast areas [ 23 , 26 ]. In the present study, due to the lack of beam hardening artifacts of root filling material, we employed a voxel size of 0.3mm. Shin *et al.* [ 28 ], using Root ZX EAL in locating root perforations, found that liquid embedding media such as saline and NaOCl are more accurate than the gel types. Another study also reported that the accuracy rate was dependent on the embedding media and apex locator type [ 24 ]. Here, we used saline with Root ZX, but using other media and EALs might change the results, which should be investigated in the future studies. In addition, future studies should examine different EALs and CBCT machines to study other positions, sizes, and types of perforations.

## Conclusion

In this ex vivo study, the sensitivity of CBCT for detecting root perforations and the negative predictive value of the test were higher than that of EAL, while the positive predictive value of EAL was higher. Hence, CBCT imaging can be preferred for diagnosis of root perforations regarding its reliable diagnostic outcome.

## Conflict of Interest

The authors declare that they have no conflict of interest.
